# Sarcoidosis presenting with facial swelling (Heerfordt syndrome)

**DOI:** 10.1002/ccr3.1741

**Published:** 2018-09-05

**Authors:** Satish Maharaj, Megan Brown, Karan Seegobin, Carmen Isache

**Affiliations:** ^1^ Department of Internal Medicine University of Florida College of Medicine Jacksonville Florida; ^2^ Department of Pathology University of Florida College of Medicine Jacksonville Florida

**Keywords:** Heerfordt syndrome, parotid swelling, sarcoidosis

## Abstract

Sarcoidosis is one of the “great masqueraders” of medicine and can present with atypical facial swelling. Imaging and biopsy confirm the diagnosis.

## 1 CASE PRESENTATION AND DISCUSSION

**Figure 1:**
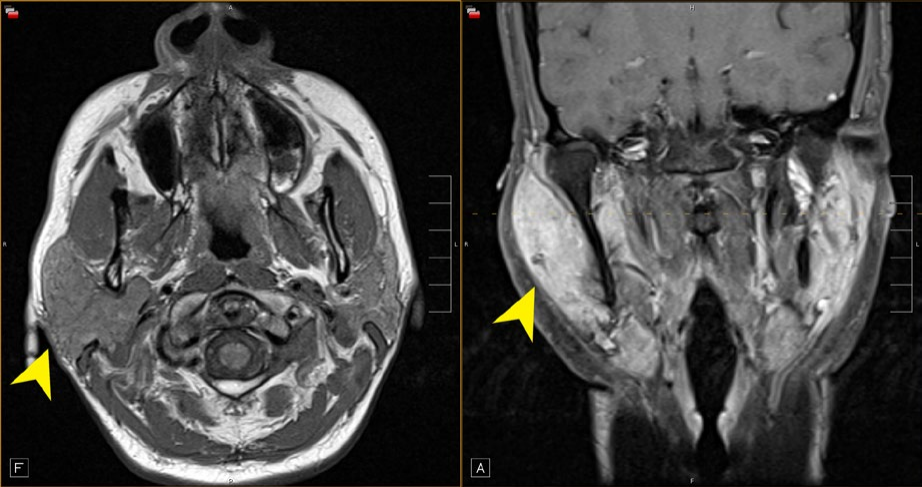
MRI revealed asymmetric enlargement of the right parotid gland involving both superficial and deep lobes

**Figure 2:**
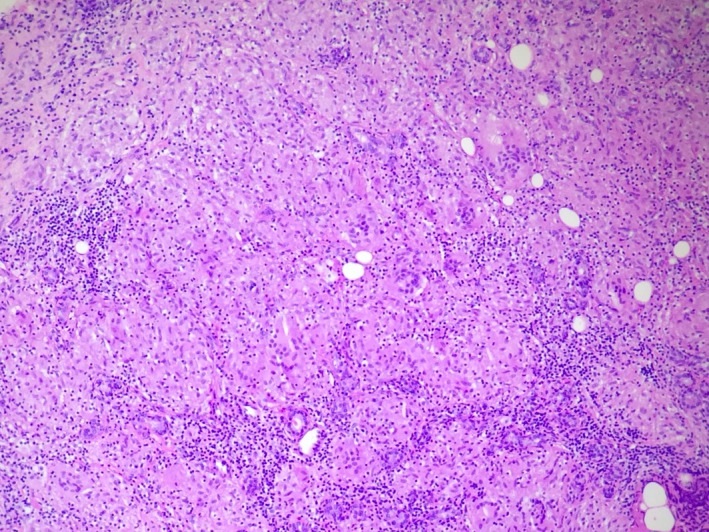
H&E, 100× magnification, demonstrates a non‐necrotizing granuloma composed of confluent nodular configuration of histiocytes and foreign body giant cells with intermixed lymphocytes and salivary ducts

**Figure 3:**
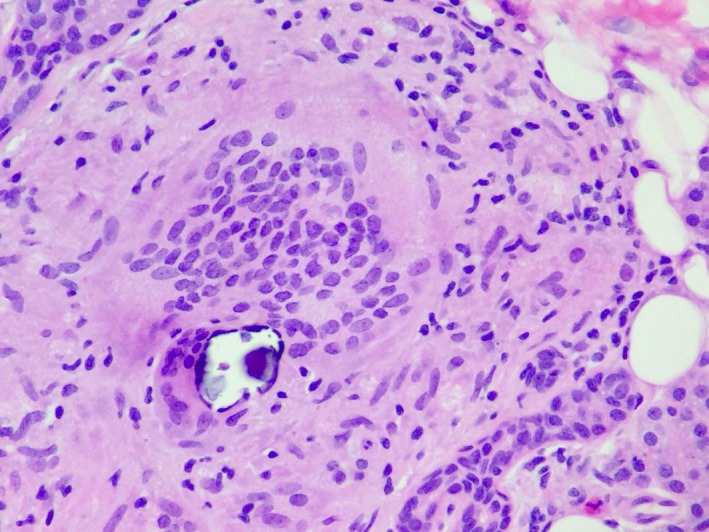
H&E, 400× magnification, demonstrates a foreign body giant cell with an inclusion body (Schaumann body) present, a finding consistent with sarcoidosis in the correct clinical setting

A 43‐year‐old female presented with a 3‐month history of right‐sided facial swelling associated with dry eyes, dysphagia with dry foods, fevers, and weight loss. On examination, there was right‐sided facial swelling, nontender. Serology for tuberculosis, human immunodeficiency virus, antinuclear, anti‐Ro, and anti‐La antibodies was all negative. A chest radiograph showed bilateral hilar adenopathy. MRI (Figure 1) revealed asymmetric right parotid enlargement involving both superficial and deep lobes. The parotid tail was excised, demonstrating non‐necrotizing granulomas (Figure 2). Closer examination (Figure 3) showed a foreign body giant cell with Schaumann body present. These findings were consistent with sarcoidosis, presenting as Heerfordt syndrome.

Sarcoidosis is a granulomatous disorder of unknown etiology and one of the “great masqueraders” of medicine. Although most patients have thoracic involvement, more than half do not have respiratory symptoms at presentation.^1^ Up to 30 percent of patients present with extrathoracic manifestations.^1^ Heerfordt syndrome was first described by the Danish ophthalmologist Christian Heerfordt in 1909, and the association with sarcoidosis was made by the Swedish internist Jan Waldenstro¨m in 1937.^2^ Due to its rarity, the exact prevalence is unknown and physicians should include the disease in the differential of parotid swelling. As in this case, chest radiography is helpful and definitive diagnosis can be made by histopathology.

## CONFLICT OF INTEREST

None declared.

## AUTHORSHIP

CI and SM: conceived the idea for the study. MB and KS: collected data on the case. All authors were involved in patient care. SM: wrote the first draft of the manuscript. All authors provided input, reviewed, and approved the final version of the manuscript.

## CONSENT

Informed consent was obtained from the patient for publication of this report.
